# *Ex vivo*^18^O-labeling mass spectrometry identifies a peripheral amyloid β clearance pathway

**DOI:** 10.1186/s13024-017-0152-5

**Published:** 2017-02-20

**Authors:** Erik Portelius, Niklas Mattsson, Josef Pannee, Henrik Zetterberg, Magnus Gisslén, Hugo Vanderstichele, Eleni Gkanatsiou, Gabriela A. N. Crespi, Michael W. Parker, Luke A. Miles, Johan Gobom, Kaj Blennow

**Affiliations:** 1 Institute of Neuroscience and Physiology, Department of Psychiatry and Neurochemistry, The Sahlgrenska Academy at University of Gothenburg, Mölndal, Sweden; 2 Clinical Neurochemistry Laboratory, Sahlgrenska University Hospital, University Hospital, SE-431 80 Mölndal, Sweden; 3 Clinical Memory Research Unit, Faculty of Medicine, Lund University, Lund, Sweden; 4 Department of Neurology, Skåne University Hospital, Lund, Sweden; 5 Department of Molecular Neuroscience, UCL Institute of Neurology, Queen Square, London, UK; 6 Department of Infectious Diseases, Institute of Biomedicine, the Sahlgrenska Academy, University of Gothenburg, Gothenburg, Sweden; 7 ADx Neurosciences, Gent, Belgium; 8 St. Vincent’s Institute of Medical Research, Fitzroy, VIC Australia; 9 Department of Biochemistry and Molecular Biology, Bio21 Molecular Science and Biotechnology Institute, University of Melbourne, Parkville, VIC Australia

**Keywords:** Amyloid β, Mass spectrometry, Stable isotope labeling, Cerebrospinal fluid, Insulin-degrading enzyme

## Abstract

**Background:**

Proteolytic degradation of amyloid β (Aβ) peptides has been intensely studied due to the central role of Aβ in Alzheimer’s disease (AD) pathogenesis. While several enzymes have been shown to degrade Aβ peptides, the main pathway of Aβ degradation *in vivo* is unknown. Cerebrospinal fluid (CSF) Aβ42 is reduced in AD, reflecting aggregation and deposition in the brain, but low CSF Aβ42 is, for unknown reasons, also found in some inflammatory brain disorders such as bacterial meningitis.

**Method:**

Using ^18^O-labeling mass spectrometry and immune-affinity purification, we examined endogenous proteolytic processing of Aβ in human CSF.

**Results:**

The Aβ peptide profile was stable in CSF samples from healthy controls but in CSF samples from patients with bacterial meningitis, showing increased leukocyte cell count, ^18^O-labeling mass spectrometry identified proteolytic activities degrading Aβ into several short fragments, including abundant Aβ1–19 and 1–20. After antibiotic treatment, no degradation of Aβ was detected. In vitro experiments located the source of the proteolytic activity to blood components, including leukocytes and erythrocytes, with insulin-degrading enzyme as the likely protease. A recombinant version of the mid-domain anti-Aβ antibody solanezumab was found to inhibit insulin-degrading enzyme-mediated Aβ degradation.

**Conclusion:**

^18^O labeling-mass spectrometry can be used to detect endogenous proteolytic activity in human CSF. Using this technique, we found an enzymatic activity that was identified as insulin-degrading enzyme that cleaves Aβ in the mid-domain of the peptide, and could be inhibited by a recombinant version of the mid-domain anti-Aβ antibody solanezumab.

**Electronic supplementary material:**

The online version of this article (doi:10.1186/s13024-017-0152-5) contains supplementary material, which is available to authorized users.

## Background

The significance of proteolytic degradation products of proteins and peptides in the central nervous system (CNS) relate not only to their potential use as biomarkers of disease activity, but also to the understanding of disease mechanisms. Amyloid β (Aβ) peptides are intensely studied due to their central role in Alzheimer’s disease (AD) [18, 19, 26]. Aβ is produced from neurons by sequential cleavage of amyloid precursor protein (APP) by the β- and γ-secretases [5], and released into the interstitial fluid of the brain, which communicates freely with the cerebrospinal fluid (CSF). Aβ is cleared from the brain by several pathways including both extra- and intra- cellular proteolytic degradation, resulting in the production of a large number of different peptides found in brain tissue and CSF [37]. Aβ is also cleared across the blood-brain barrier into the blood, and by CSF absorption into the circulatory or lymphatic system (for review see [49]). While Aβ is effectively degraded in the blood, it is not clear to which extent it is degraded in the interstitial fluid or CSF [24].

AD patients have reduced CSF levels of the 42 amino acid long peptide Aβ42 [6, 36], but low CSF Aβ concentration has also been found in infectious or inflammatory CNS disorders, for example in patients suffering from bacterial meningitis, neuroborreliosis, multiple sclerosis and HIV with CNS engagement [17, 27, 28, 33, 46, 50]. In bacterial meningitis, low CSF Aβ42 normalizes after proper antibiotic treatment, and it has been hypothesized that the decrease in CSF Aβ42 during the acute infection may be caused by impaired clearance of Aβ from the brain, and not related to the inflammation in itself [46]. Another explanation may be increased activities of APP and Aβ metabolizing enzymes with inflammation.

In the current work, we explore for the first time the use of proteolytic ^18^O labeling and mass spectrometry (MS) to detect endogenous proteolytic activity in human CSF. CSF was collected from patients with bacterial meningitis before and after successful treatment with antibiotics. The samples were incubated with ^18^O-enriched water and subjected to immunoaffinity purification of Aβ followed by MS to measure the degree of incorporation of ^18^O into proteolytically produced Aβ peptides, enabling determination of the relative amount of peptides formed by endogenous proteolytic activity in the CSF. Using this approach, we identified products of a specific Aβ-degrading activity in CSF from meningitis patients in the acute phase. This activity, likely derived from blood cell components in the CSF, was identified to arise from insulin-degrading enzyme (IDE), possibly representing a major degradation pathway of Aβ. We also show that this proteolytic activity can be blocked by a recombinant version of the mid-domain anti-Aβ antibody solanezumab that has an epitope covering the discovered cleavage site on Aβ.

## Methods

### Study subjects

CSF was provided from the Clinical Neurochemistry Laboratory, Mölndal, Sweden. The samples were surplus from the clinical routine, used after de-identification. The samples were taken from patients with neurochemical signs of meningitis including increased CSF cell counts and CSF/serum albumin ratio (see Table 1 for demographics). Blood samples were obtained from healthy volunteers. Erythrocyte concentrate (225–340 ml prepared from 400 to 450 ml whole blood) was provided by the Blood Center at the Sahlgrenska University Hospital. The study was approved by the local Institutional Review Boards and was performed in accordance with the ethical standards laid down in the 1964 Declaration of Helsinki.

**Table 1:** Clinical characteristics in the study groups. Cell count is n/μl CSF (reference range < 4 /μl). Albumin is mg/l (reference range < 400 mg/l)

Patient no.	Disease phase	CSF cell count		CSF Albumin
		Polynuclear	Mononuclear	
1	Acute phase	31	43	560
	After treatment	0	2	259
2	Acute phase	445	80	3132
	After treatment	0	15	215
3	Acute phase	364	80	2546
	After treatment	2	37	143
4	Acute phase	310	12	665
	After treatment	46	4	211
5	Acute phase	9000	900	1650
	After treatment	missing	missing	1710
6	Acute phase	3400	744	missing
	After treatment	17	60	361

### Proteolytic ^18^O-labeling in CSF

The CSF samples (1 ml) were pH-stabilized by addition of 1 M HEPES buffer, pH 7.4 to a final concentration of 100 mM. H_2_^18^O (Sigma) were added to the samples at a 1:1 ratio (v/v), and the samples were incubated for 24 h at room temperature. The samples were stored at −80 °C pending analysis.

### Aβ immunoaffinity purification

Aβ peptides were immunoprecipitated using Aβ-specific antibodies coupled to magnetic beads as described previously [38]. Briefly, 4 μg of the anti-Aβ antibodies 6E10 and 4G8 (Signet Laboratories, Dedham, MA, USA) were separately added to 50 μL each of magnetic Dynabeads M-280 Sheep Anti-Mouse IgG (Invitrogen, Carlsbad, CA, USA). The 6E10 and 4G8 antibody-coated beads were mixed and added to the CSF samples to which 0.025% Tween20 in phosphate-buffered saline (pH 7.4) had been added. After washing, the Aβ peptides were eluted using 100 μL 0.5% formic acid.

### Mass spectrometry

Mass spectrometry was performed using a matrix-assisted-laser-desorption/ionizationtime-of-flight/time-of-flight (MALDI TOF/TOF) instrument (UltraFleXtreme, Bruker Daltonics, Bremen, Germany). Samples were prepared as described previously [38].

### Test of Aβ degrading activity in CSF containing trace amounts of blood

The isotope labeled Aβ peptide Aβ1-40 Arg^13^C^15^N (Anaspec, Inc., San Jose, USA) was dissolved in dimethylsulfoxide (DMSO, Aldrich) to a concentration of 1 mg/mL. The stock solution was aliquoted and immediately stored at −20 °C. Before use the labeled peptide was diluted and mixed to a final concentration of 0.8 μM. A 10-μL aliquot of the peptide solution was added to 930 μL CSF to which blood was added at different final concentrations (0, 0.05, 0.5, 5% (v/v)) followed by incubation in room temperature overnight at room temperature. IP-MS was conducted as described above.

### In vitro test of Aβ1-40 degradation by IDE

An aliquot (10 μL) of a 0.8 μM solution of the isotopic labeled Aβ1-40 (Arg^13^C^15^N) was added to 980 μL CSF and incubated 5 min in room temperature. Recombinant human IDE (R&D systems) was diluted in 50 mM ammonium bicabonate to 23.6 ng/mL and 5.9 ng/mL. 10 μL of each solution was added to two separate pre-incubated CSF samples and left over night in room temperature. Immunoprecipitation and mass spectrometry were conducted as described above.

### Inhibition of IDE activity by insulin

The inhibition of IDE activity was addressed by adding isotopic labeled Aβ1-40 (Arg^13^C^15^N, end concentration 10 ng/mL) to CSF followed by addition of recombinant human IDE (0.1, 1 or 10 μM, Sigma) and 0.5% human blood. The samples were incubated overnight at room temperature. Immunoprecipitation and mass spectrometry were conducted as described above.

### Identification of Aβ degrading activity in leukocytes, thrombocytes, and erythrocytes

Leukocytes and thrombocytes were isolated from 10 mL blood as described previously [13, 35]. The cells were resuspended in 500 μL ultra-pure water, lysed by four freeze/thaw cycles and finally centrifuged 10 min (+4 °C, 31,000 x g). Erythrocytes were prepared using a Reveos automated blood component extractor.

To 100 μL ammonium bicarbonate, spiked with 5 pmol/μL isotopic labeled Aβ1-40, 5 μL leukocytes, thrombocytes, or erythrocytes were added, followed by incubation overnight at room temperature. Immunoprecipitation and mass spectrometry were conducted as described above.

### The effect of antibodies on Aβ degradation

To 1 ml PBS containing isotopic labeled Aβ1-40 (0.016 μM) 4 μg of each of the recombinant antibodies solanezumab, bapineuzumab and crenezumab were added [8, 53], followed by incubation for 1 h at room temperature. The antibodies were expressed and purified as previously described [53]. Each sample was spiked with serum (5%, end concentration) followed by incubation overnight at room temperature. The samples were placed in a 100 °C heating block for five minutes to disrupt potential antibody/Aβ complexes, and subsequently centrifuged for 10 min at 4 °C (31.000 x g). The supernatants were transferred to new tubes and Aβ peptides were immunoprecipitated and analyzed by mass spectrometry as described above.

### Isotope distribution calculations

Theoretical peptide isotope distributions with and without incorporation of one ^18^O atom at 50% abundance were calculated using the software Isotope Distribution Calculator v 0.3 [21]. The fractional abundance of Aβ peptides, produced by proteolysis during the incubation period, was calculated by linear regression analysis according to Mirgorodskaya et al. [31], based on the observed isotope distributions of the detected peptides.

## Results

### Proteolytic ^18^O labeling detects aberrant Aβ processing in the CSF during acute bacterial meningitis

**Fig. 1:**
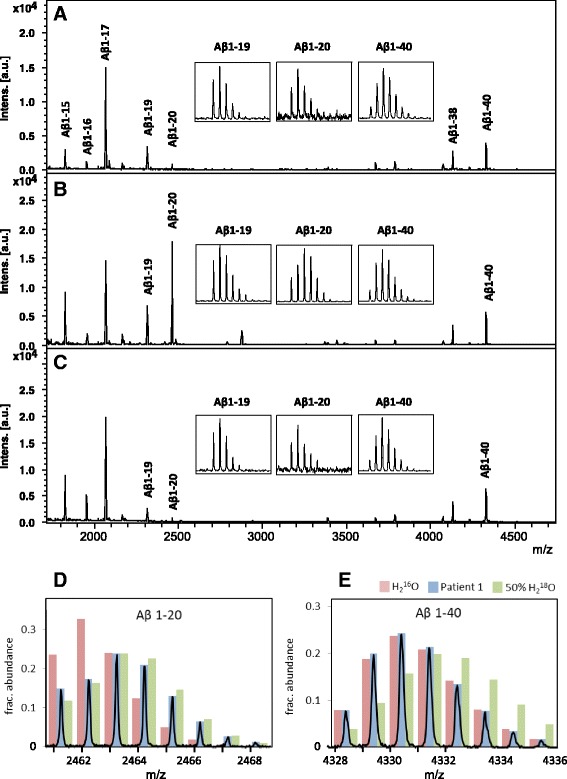
MALDI-TOF MS CSF Aβ peptide patterns of **a** a non-symptomatic control, **b** Patient 1 in the acute phase of BM and **c** after antibiotic treatment. The recorded isotope patterns of **d** Aβ 1–20 and **e** Aβ 1–40 in acute BM are shown in *blue* bars, and the expected isotope patterns of the peptides without ^18^O incorporation and with one oxygen atom exchanged for ^18^O at 50% abundance is indicated by *red* and *green* bars, respectively

Initial experiments had shown that a patient in the acute phase of bacterial meningitis displayed an abnormal Aβ peptide pattern (Additional file 1: Figure S1). To test for Aβ/APP proteolytic processing in the CSF, H_2_^18^O was added to CSF samples at a volume ratio of 1:1, followed by incubation at room temperature for 24 h. The samples were then subjected to Aβ immunoprecipitation, and analyzed by MALDI-TOF MS [39]. In control samples (Fig. 1a), a normal Aβ peptide pattern was observed, as previously reported [39], and the isotope distributions of the Aβ peptide signals did not differ from samples not incubated with H_2_^18^O (data not shown), indicating that there is no proteolytic activity affecting the Aβ peptides in normal CSF. In contrast, in CSF samples from patients (Table 1) in the acute phase of bacterial meningitis (Fig. 1b), a strong signal at *m*/*z* 2461.17 (monoisotopic mass) was present, corresponding to Aβ1-20 as verified by MS/MS (data not shown). In three of the patients the appearance of this signal was accompanied by an apparent decrease in Aβ1-40 (*m*/*z* 4328.16). In CSF samples from bacterial meningitis patients taken after antibiotic treatment (Fig. 1c), the normal Aβ peptide pattern was restored. In addition, two unidentified compounds at *m*/*z* 3369.45 and 3440.50 were detected in three of the patients in the acute phase. Peaks at these *m*/*z* values were also detected in blood-containing samples (see below), indicating blood as their likely source of origin.

Incubation of the samples in H_2_^18^O results in the incorporation of ^18^O at the C-terminal carboxy group of peptides formed by proteolysis, resulting in a molecular mass shift of +2 or + 4 Da. The mix of labeled/unlabeled peptides resulting from partial labeling and unlabeled peptide formed prior to addition of H_2_^18^O manifests in the mass spectra as a broadening of the natural isotopic peak distribution. In Fig. 1d and e, the red bars represent the theoretical isotope distributions of Aβ1-20 and Aβ1-40 without incorporation of ^18^O. The green bars indicate the theoretical distributions expected from incorporation of one ^18^O at 50% abundance, representing full incorporation under the give experimental conditions. By comparing the theoretical isotope distributions with the recorded isotopic peak distributions, the fractional abundance of proteolytically produced Aβ1-20 and 1-40 was calculated by linear regression analysis [31] for all patients before and after treatment (Table 2). In five out of six patients the fractional abundance was close to one, i.e., the recorded isotope distribution of Aβ1-20 corresponded to the calculated isotope distribution of the peptide with exchange of one ^16^O oxygen atom for ^18^O at 50% abundance (Fig. 1d), implying that this peptide was mostly formed by proteolysis in CSF during the experiment. In contrast, the fractional abundance of ^18^O-Aβ1-40 was close to zero, i.e., its isotope distribution indicates no incorporation of ^18^O (Fig. 1e). Thus the Aβ1-40 peptide was not formed by proteolytic cleavage in the CSF.

**Table 2:** Fraction Aβ 1–20 formed during incubation of CSF at room temperature for 24 h, calculated based on the altered peptide isotope distribution due to the incorporation of H_2_^18^O

	Aβ1-20		Aβ1-40	
Patient #	Before treatment	After treatment	Before treatment	After treatment
1	1.14	ND	−0.03	0.05
2	1.10	ND	0.12	−0.10
3	1.10	ND	0.16	0.04
4	0.59	0.15	0.18	−0.16
5	1.02	0.27	ND	0.04
6	1.06	0.22	−0.08	0.21

### Blood contamination in the CSF produces *acute bacterial meningitis* -like Aβ degradation

**Fig. 2:**
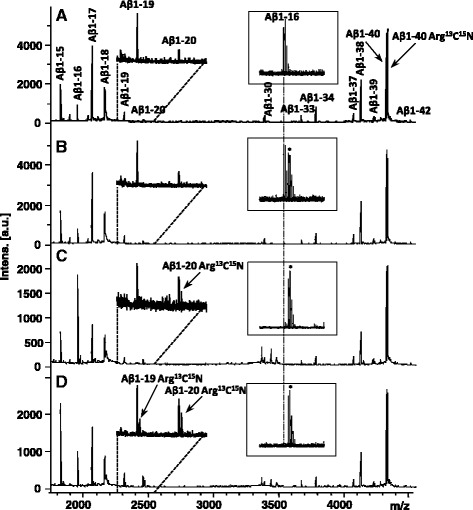
MALDI-TOF mass spectra of the CSF Aβ pattern after incubation with **a** 0%, **b** 0.05%, **c** 0.5% and **d** 5% human blood. The inset shows the degradation of Aβ1-16, and the vertical dashed line indicates its monoisotopic *m*/*z*. * represents an unidentified peak, of three Da higher mass than the monoisotopic peak of Aβ1-16. The Arg^13^C^15^N will appear as a mass shift of 10 Da relative the endogenous peptide as seen for Aβ1-19 and Aβ1-20

Considering the possibility that the proteoloytic activity found in bacterial meningitis CSF represented an enzymatic activity derived from blood we next incubated CSF from a healthy individual, spiked with stable isotope labeled Aβ1-40 Arg^13^C^15^N, with different percentages of fresh blood (v/v). The addition of blood produced an Aβ peptide pattern similar to that of patients with acute phase bacterial meningitis, with increased levels of Aβ1-19 and Aβ1-20 (Fig. 2). Degradation of Aβ1-40 was verified by the detection of Aβ1-19 Arg^13^C^15^N and Aβ1-20 Arg^13^C^15^N from the added stable isotope labeled Aβ1-40 Arg^13^C^15^N (Fig. 2c-d). Other proteolytic activity degraded endogenous Aβ1-16 further, as is evident by the decreasing abundance of the peptide signal (Fig. 2, *inset*). In the sample incubated with 0.5% blood in the CSF (Fig. 2d), endogenous Aβ1-16 was undetectable.

**Fig. 3:**
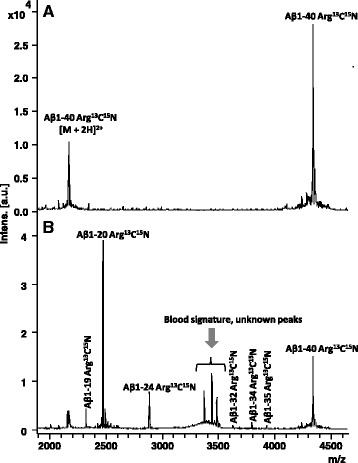
Mass spectra of PBS spiked with Aβ1-40 Arg^13^C^15^N, incubated overnight in the absence (**a**) and presence (**b**) of 0.5% blood. In **b**, Several Aβ degradation products are detected as well as a cluster of signature peaks from blood in the mass range 3300 – 3600 Da, the identities of which are unknown

These proteolytic activities were not dependent on CSF-specific factors, since a similar degradation pattern was observed when replacing CSF with PBS buffer in the experiment above, resulting in proteolysis of the stable isotope labeled Aβ1-40 into similar shorter peptides as observed in CSF (Fig. 3).

### The Aβ degrading activity is present in blood components

**Fig. 4:**
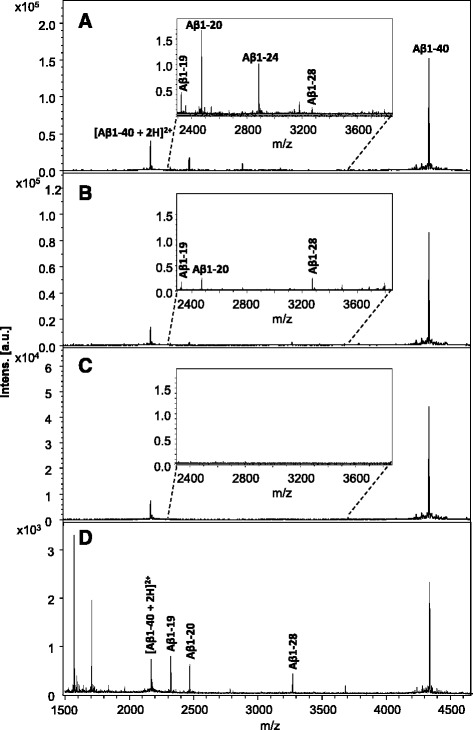
Mass spectra of Aβ1-40 proteolytic products after incubation with **a** leukocytes, **b** thrombocytes, **c** plasma without thrombocytes, and **d** erythrocytes

To pinpoint the origin of the observed proteolytic activity, blood was fractionated into leukocytes, thrombocytes, thrombocyte-free plasma, and erythrocytes. The fractions were spiked with Aβ1-40, incubated as described above, and analyzed by IP-MALDI MS (Fig. 4). While minor degradation was observed in the thrombocyte fraction, the major effect was observed in the leukocyte and erythrocyte fractions where Aβ1-40 was clearly degraded into shorter Aβ peptides, preferentially Aβ1-20. Further experiments showed some Aβ1-40 degradation after incubation with 5% serum but almost no degradation in the presence of plasma (Additional file 2 Figure S2).

### IDE is a candidate protease for CSF Aβ degradation in *acute bacterial meningitis* patients

**Fig. 5:**
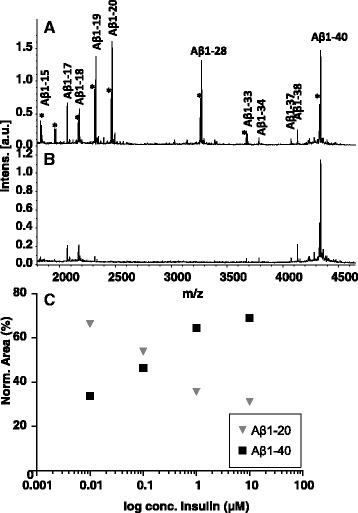
Mass spectra of CSF spiked with Aβ1-40 and its proteolytic products, after incubation with **a** IDE, and **b** IDE and insulin. **c** Scatter plot of Aβ1-20 and Aβ1-40 versus log insulin conc. in PBS spiked with Aβ1-40 and IDE after incubation with insulin at different concentrations

It is known that the protease insulin-degrading enzyme (IDE) cleaves Aβ between residues 19–20 and 20–21 [34]. To test if the observed Aβ degradation was caused by IDE, stable isotope labeled Aβ1-40 Arg^13^C^15^N and IDE were added to CSF from healthy individuals, followed by incubation and subsequent IP-MALDI MS analysis. Massive degradation of Aβ was observed, the most prominent cleavage products being Aβ1-19, Aβ1-20 and Aβ1-28 (Fig. 5a, b).

To investigate if the IDE-mediated proteolytic activity could be inhibited, we added increasing amounts of insulin to aliquots of PBS spiked with Aβ1-40 Arg^13^C^15^N. As hypothesized, the abundance of Aβ1-20 decreased with increasing insulin concentration, while Aβ1-40 increased (Fig. 5c).

### An anti-Aβ antibody blocks degradation of Aβ in serum

**Fig. 6:**
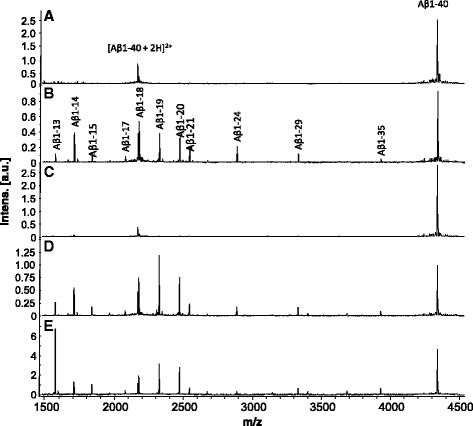
Mass spectra of Aβ1-40 and its proteolytic products in PBS (**a**), after incubation with 5% serum with **b** no antibody, **c** in the presence of Solanezumab, **d** Crenezumab, and **e** Bapineuzumab

It has previously been shown that anti-amyloid treatment using some Aβ-specific monoclonal antibodies, such as the mid-domain anti-Aβ antibody solanezumab [14, 45] and the C-terminal (Aβ X-40) antibody ponezumab [9, 22] increases plasma Aβ concentration up to thousand-fold compared to physiological levels (For review, see [7]). It has been suggested that this increase may be caused by some antibodies blocking the cleavage sites of proteases that normally degrade Aβ [7]. To explore this hypothesis, we tested the effect of recombinant versions of the antibodies solanezumab [14], bapineuzumab [47], and crenezumab [53]. The anti-Aβ antibodies were incubated together with Aβ1-40 in the presence of 5% serum (Fig. 6). While incubation in 5% serum in the absence of antibody gave rise to multiple shorter Aβ peptides including Aβ1-19 and Aβ1-20 (Fig. 6b), the proteolytic degradation was effectively abrogated when the sample was pre-treated with recombinant solanezumab antibody (Fig. 6c). In contrast, incubation in the presence of recombinant crenezumab (Fig. 6d) and bapineuzumab (Fig. 6e) antibodies did not block the degradation of Aβ.

## Discussion

We report on an enzymatic ^18^O labeling-mass spectrometry assay for determination of the relative amount of the peptides formed by endogenous proteolytic activity. ^18^O-labeling has previously been used for determination of proteins’ C-termini [41], for the identification of cross-linked peptides in proteolytic peptide mixtures [1], to assist the interpretation of fragment ion mass spectra [43], for generating calibrators for mass spectrometric protein quantification [31], and for differential analysis of protein mixtures [2, 30, 52, 55]. In a recent paper, ^18^O-labeling was used to monitor proteolytic degradation during sample preparation of mouse brain [48]. Here we show for the first time that ^18^O-labeling can also be used to detect endogenous proteolytic activity associated with disease. While the current study focuses on the processing of Aβ, the method may be applied to detect proteolytic processing events leading to the formation of any peptide and could thus be a universal tool to assess the integrity of CSF peptides over time; an important aspect in the development of biomarkers.

In bacterial meningitis, activated neutrophils are activated and released.﻿ Using ^18^O-labeling, we found that proteolytic activity in the CSF from patients with bacterial meningitis in the acute phase degrades full-length Aβ peptides into shorter forms, with Aβ1-19, Aβ1-20 and Aβ1-24 being prominent cleavage products. In CSF samples from bacterial meningitis patients taken after antibiotic treatment, the activity was abrogated, also concurring previous data showing that Aβ1-42 in CSF is normally stable over time, even at room temperature [4].

Spiking CSF and solutions containing Aβ with blood produced the similar Aβ fragments as in CSF from patients with bacterial meningitis, suggesting a blood component as the likely source of the observed activity. Similar activity was observed when Aβ1-40 was incubated with serum. The lesser activity observed in plasma likely reflects the presence of EDTA in the collection tubes, which previously has been shown to inhibit IDE proteolytic activity [25]. Further spiking experiments showed that the proteolytic activity resided in leukocytes as well as erythrocytes. IDE activity has previously been associated with leukocytes [42] and erythrocytes [44]. In-vitro experiments have shown that IDE can cleave Aβ between position 19 and 20, and 20 and 21 in the amino acid sequence [29], suggesting it as a possible mediator of the observed activity. We also detected prominent Aβ1-19 and Aβ1-20 signals in CSF samples spiked with IDE (Fig. 5). There are some differences between the results obtained with PBS spiked with blood. For example, a prominent Aβ1-28 signal is present in CSF incubated with IDE but absent in Aβ1-40 incubated with blood. This may be attributed to other proteolytic activities in blood that further degrade Aβ1-28 to shorter Aβ species. Similarly, Aβ1-24 in an Aβ1-40 sample spiked with blood may be the product of other blood-derived proteases.

IDE is a zinc-metallopeptidase which has been implicated in several prevalent diseases including Type 2 diabetes mellitus and AD [12]. IDE has been found to degrade and influence brain Aβ in experimental animal models [3, 23, 32, 54] and it has also been shown to be present in serum [25] and CSF [40]. In the present experiments, no formation of Aβ1-19 or Aβ1-20 was observed in CSF samples from patients taken after antibiotics treatment with normalized leukocyte counts. Furthermore, it has previously been shown that Aβ1-42 in CSF is normally stable over time, even at room temperature [4].

Our results indicate that cleavages at positions Aβ19 and Aβ20 represents a significant pathway for physiological Aβ degradation in blood, and that these cleavages may be mediated by IDE. Since IDE-deficient mice show accumulation of Aβ protein [15], hyperinsulinemia and glucose intolerance enhancing or emulating the activity of IDE may lower the Aβ burden and may be of interest to pursue as an AD-treatment.

The dramatic increase observed in plasma Aβ, in response to treatment with Aβ-specific monoclonal antibodies [14, 45] has been attributed to increased clearance of soluble Aβ from the brain into the periphery. According to the peripheral sink hypothesis, the therapeutic antibody changes the equilibrium for Aβ between the brain and plasma, with increased clearance from the brain and a resulting increase in plasma Aβ levels [11]. However, studies in mouse models, in which the peripheral level of Aβ was decreased by either by reducing production or increasing degradation of Aβ in the periphery, failed to show any effect on Aβ levels in the brain [16, 20, 51]. An alternative hypothesis is that some anti-Aβ antibodies cause a prolonged half-life of Aβ peptides in the bloodstream by blocking protease cleavage sites [7].

Here we show that treatment with a recombinant version of solanezumab protects Aβ from degradation in serum. In contrast, bapineuzumab or crenezumab did not affect the Aβ peptide profiles, with the exception that Aβ1-13 seems to increase. This difference may be due to the much lower affinity of crenezumab [10] and bapineuzumab than solanezumab for Aβ. The pathophysiological relevance of the observations needs to be determined in future studies.

## Conclusions

In the present study, we show that a technique based on enzymatic ^18^O labeling-mass spectrometry is useful for identifying and determining the relative amount of peptides formed by endogenous proteolytic activity in human CSF. Using this technique we found an enzymatic activity in blood leukocytes and erythrocytes that was identified as IDE that cleaves Aβ in the mid-domain of the peptide, and could be inhibited by a recombinant version of the mid-domain anti-Aβ antibody solanezumab. If, as these results suggest, the increase in plasma Aβ upon treatment with some Aβ-specific antibodies is caused by blocking the protease cleavage site, that is that the higher affinity antibody (solanezumab) reduces IDE processing of the peptide, it should be considered that therapeutic antibodies may in fact interfere with Aβ clearance by stabilizing Aβ peptides.

## Additional files

Additional file 1: Figure S1. MALDI-TOF MS CSF Aβ peptide patterns of a patient (A) in the acute phase of BM and (B) after antibiotic treatment. (PPTX 74 kb)
Additional file 2: Figure S2. Effect of serum and plasma on Aβ degradation. PBS spiked with (A) Aβ1-40 Arg^13^C^15^N and 5% serum and (B) Aβ1-40 Arg^13^C^15^N and 5% plasma. (PPTX 58 kb)
