# Supplementary material for: Ex vivo18O-labeling mass spectrometry identifies a peripheral amyloid β clearance pathway
Source: Mol Neurodegener. 2017 Feb 20;12:18. doi: 10.1186/s13024-017-0152-5 (PMC5317049; doi:10.1186/s13024-017-0152-5)
Supplement: Additional file 1: Figure S1. — MALDI-TOF MS CSF Aβ peptide patterns of a patient (A) in the acute phase of BM and (B) after antibiotic treatment. (PPTX 74 kb) [file 13024_2017_152_MOESM1_ESM.pptx]

## Slide 1
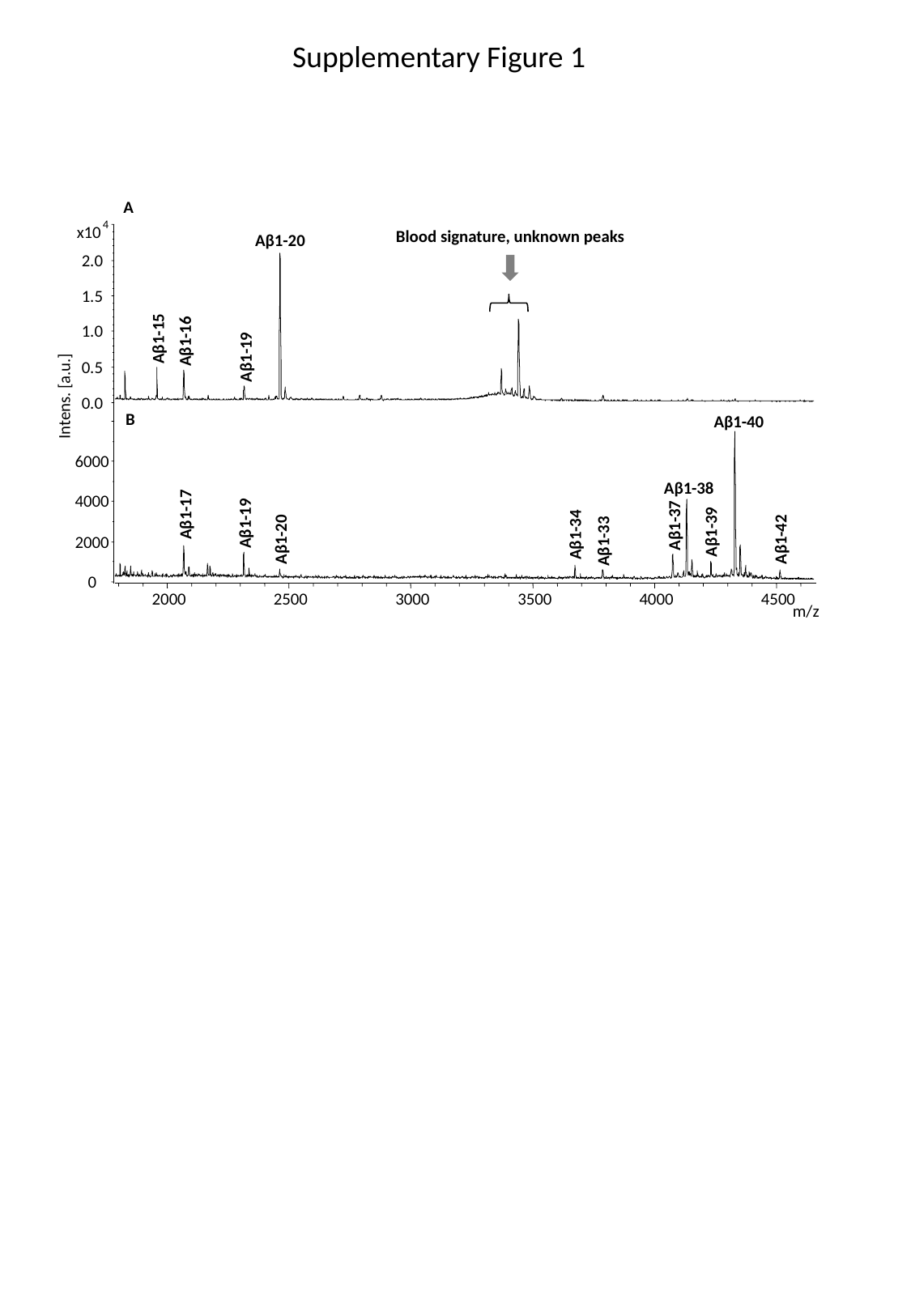

Supplementary Figure 1
A
4
Blood signature, unknown peaks
x10
Aβ1-20
2.0
1.5
1.0
Aβ1-15
Aβ1-16
Aβ1-19
0.5
Intens. [a.u.]
0.0
B
Aβ1-40
6000
Aβ1-38
4000
Aβ1-17
Aβ1-19
Aβ1-37
Aβ1-39
Aβ1-34
Aβ1-20
Aβ1-42
Aβ1-33
2000
0
2000
2500
3000
3500
4000
4500
m/z
