# Supplementary material for: Ex vivo18O-labeling mass spectrometry identifies a peripheral amyloid β clearance pathway
Source: Mol Neurodegener. 2017 Feb 20;12:18. doi: 10.1186/s13024-017-0152-5 (PMC5317049; doi:10.1186/s13024-017-0152-5)
Supplement: Additional file 2: Figure S2. — Effect of serum and plasma on Aβ degradation. PBS spiked with (A) Aβ1-40 Arg13C15N and 5% serum and (B) Aβ1-40 Arg13C15N and 5% plasma. (PPTX 58 kb) [file 13024_2017_152_MOESM2_ESM.pptx]

## Slide 1
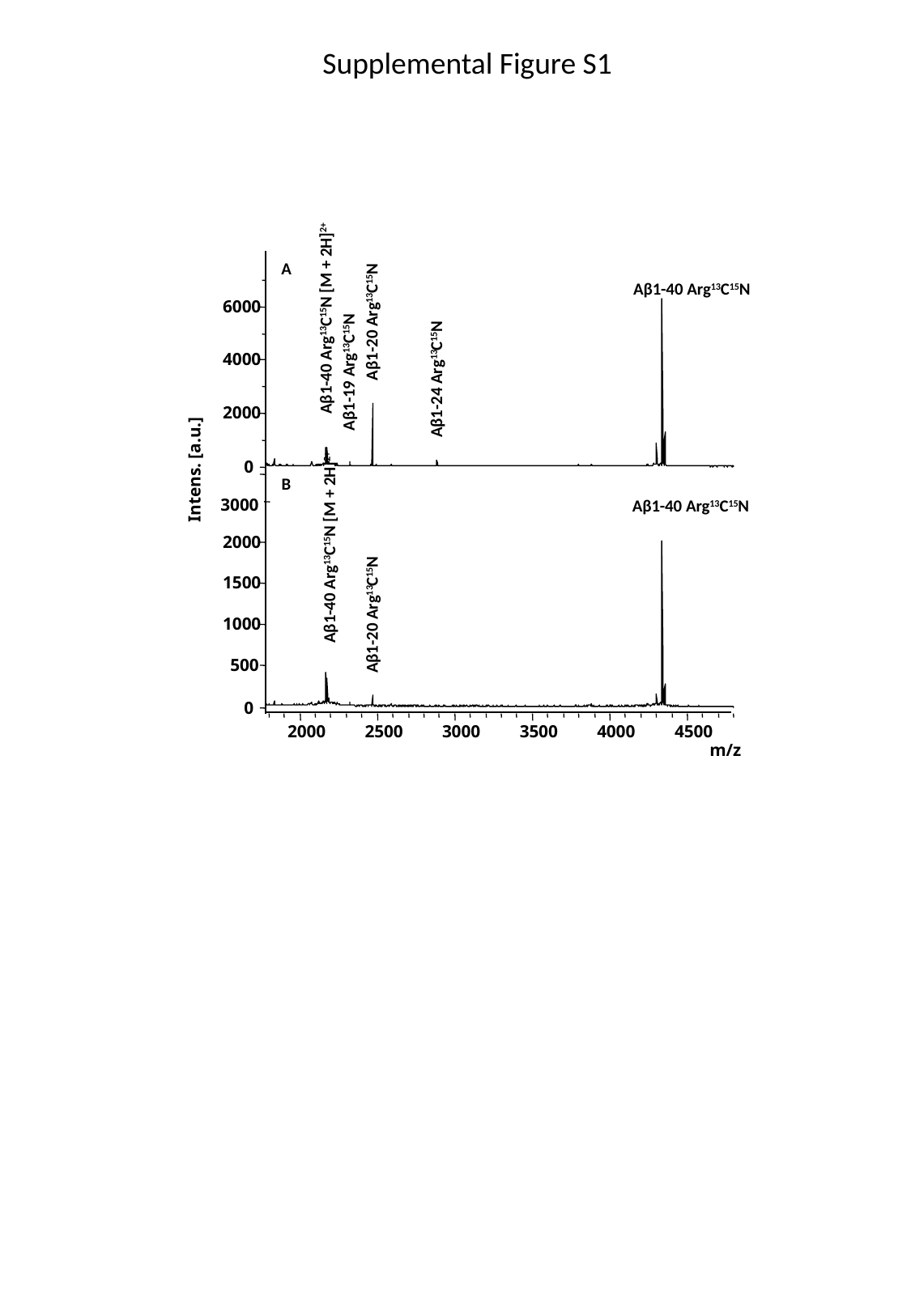

Supplemental Figure S1
A
Aβ1-40 Arg13C15N
6000
Aβ1-40 Arg13C15N [M + 2H]2+
Aβ1-20 Arg13C15N
4000
Aβ1-19 Arg13C15N
Aβ1-24 Arg13C15N
2000
0
Intens. [a.u.]
B
Aβ1-40 Arg13C15N
3000
Aβ1-40 Arg13C15N [M + 2H]2+
2000
1500
Aβ1-20 Arg13C15N
1000
500
0
2000
2500
3000
3500
4000
4500
m/z
